# Psychopathology, psychopathy, body management, and undoing in youthful parricide offenders

**DOI:** 10.1111/1556-4029.15636

**Published:** 2024-10-25

**Authors:** Wade Myers, Heng Choon (Oliver) Chan, Mark Safarik, Zain Khalid, Eleanor Vo

**Affiliations:** ^1^ Department of Psychiatry and Human Behavior Brown University Medical School Providence Rhode Island USA; ^2^ Department of Social Policy, Sociology, and Criminology University of Birmingham Birmingham UK; ^3^ Forensic Behavioral Services Fredericksburg Virginia USA; ^4^ OmaDesala Psychiatric Services Ewing New Jersey USA

**Keywords:** adolescent, body management, parricide, psychopathology, psychopathy, undoing, youth

## Abstract

This study of 21 youthful parricide offenders (YPOs) ages 11–21 examined the relationship between psychopathology, level of psychopathy, and crime scene behaviors, particularly two forms of “body management”: (1) “body movement” and (2) “body alteration.” Undoing, a form of body alteration reflecting psychological rather than practical needs, for example, to lessen guilt or symbolically reverse the killing, was present in one‐third of the sample. The sample shared common characteristics with juvenile parricide offenders overall in that most of them killed one parent, were victims of chronic child abuse, had one or more psychiatric diagnoses (PTSD was most prevalent), used a firearm, and committed the killings at the family home. Unexpectedly, YPOs who were child abuse victims were not more likely to engage in undoing behaviors than non‐abused youth, and over half of the youth with undoing behaviors had elevated psychopathy levels. Three‐quarters of youth who moved victim bodies following the murders had elevated psychopathy levels. Similarly, all three youth who transported bodies away from the crime scene had elevated psychopathy levels and conduct disorder. None of the youth with psychotic symptoms engaged in undoing behaviors, altered victim bodies, moved bodies either within or away from the home, or had an elevated degree of psychopathy; they also most often killed using bladed weapons, whereas the nonpsychotic youth usually used firearms. Studies examining the influence of mental illness and psychopathy level on parricidal behaviors by youth are scarce, and to our knowledge this is the largest study to date investigating this area.

**Highlights**

- Psychiatric disorders (95%) and child abuse (62%) were highly prevalent in this sample of YPOs.
- Findings on motivation, DSM‐5 diagnoses, psychopathy level, and body management were significant.
- Three‐quarters of youth who moved victim bodies had elevated psychopathy levels.
- Undoing was surprisingly common in these YPOs, present in one‐third of the sample.

## 1 INTRODUCTION

Parricide, the act of killing one's own parent, is a relatively rare form of murder. In the United States, parricide has consistently accounted for about 2.5% of all US homicides in recent years [1]. Similar frequencies, ranging from 1% to 5%, have been observed in other parts of the world [2, 3, 4, 5]. Most US parricides, about 80%, are committed by adults [6, 7].

Extrapolating from the above databases, juveniles (<18) commit roughly 50–100 parricides annually in the United States. The infrequency of juvenile parricides and only a small pool of investigators studying them has resulted in limited research findings to date [8]. However, a general understanding of these perpetrators has slowly come into focus. Juvenile parricides most commonly involve an abused adolescent son killing a single parent, usually the father, in the family home using a firearm [9]. The most widely referenced typology for parricide, developed by Heide and applicable to juveniles and adults, consists of three offender categories: the severely abused offender, the severely mentally ill offender, and the dangerously antisocial offender [10].

Shifting to the background for this present work, juvenile parricide studies examining the relationship between psychopathology and crime scene behaviors are sparse and consist of case reports and a couple of modest case series [11, 12]. Researchers believe that parricidal youth's psychosocial and cognitive characteristics will be reflected in their crime scene actions [10, 11, 13]. For instance, evidence of excessive violence, leaving incriminating evidence at the crime scene, and readily admitting to the crime would suggest the impulsive, affective nature of such offenses [14, 15, 16]. Myers and Vo [11], in their sample of 10 juvenile parricide offenders, found that 50% of the eight youth who were severely abused covered or wrapped their victims, and the two youth with elevated psychopathy scores (PCL scores >10) were the only offenders in the series who moved victim bodies outside the family home. Amorado et al. [12] reported the seven juvenile parricide offenders (most of whom were abuse victims) in their series positioned, moved, or transported victim bodies in one‐quarter to one‐third of the cases, whereas none were covered.

To further explore this area of inquiry, one focus of this present study was on youthful parricide offender (YPO) psychopathology and two forms of readily observed crime scene behaviors that fall under what we have termed “body management.” We further divided body management into two subtypes: (1) “body movement” and (2) “body alteration.” Every parricide offender following their murderous act is faced with deciding what to do, if anything, with the body of their parent(s). There are three general post‐murder pathways for body management pertaining to movement: (1) leaving the body as is, (2) moving it elsewhere within the general crime scene location, usually the family home, or (3) transporting it to a location away from the crime scene.

The other form of body management, body alteration, concerns any offender behavior that alters the appearance, degree of visibility, or state of the body other than moving it. Body alteration may include a myriad of actions, like covering or wrapping the face or body, cleaning it, removing or changing bloody clothes, disfiguring the face or otherwise mutilating the corpse, or setting it on fire as just a few examples. We expected that body alteration in youthful parricides will commonly manifest as the subtype referred to as “undoing” (or “symbolic reversal”), in accordance with the limited literature on this topic and empirical observations from our forensic practices. Undoing is thought to reflect the offender's psychological rather than practical needs, such as to lessen feelings of guilt and shame [11, 17, 18]. In psychodynamic terms, it can be conceptualized as the defense mechanism of “magical thinking”: the belief that one's actions can undo or at least mitigate an undesirable event and thus create psychological distance from anxiety or agony associated with the act of killing [19]. Undoing may be evident by such actions as covering the face or body of the victim or symbolically comforting them, for example, placing a pillow under their head, covering them with a blanket, repositioning them into a comfortable pose. Russell et al. [18], in their study of 975 nonrepresentative homicide offenders who were primarily adults, found that undoing behaviors occurred in only 11 (1%) of the cases, and 10 of the 12 victims in those cases were family members or intimate partners.

As alluded to above, the motivation for body alteration has other explanations besides undoing, and it may not be understood through crime scene findings alone [18, 20, 21]. For example, offenders may alter bodies because of psychosis or other mental disorders, to express persistent post‐homicide rage, or to fulfill paraphilic needs. Author WM consulted on a case of a late adolescent male with schizophrenia who murdered both of his parents while under the influence of paranoid delusions. Investigators observed that their bodies were covered in a white powdery substance. After his arrest, the defendant explained he had generously sprinkled an Asian healing powder (a folk medicine) on their bodies to restore them to life. In other parricide cases we have seen during consultation, rageful offenders have beaten victim faces to the point of being unrecognizable or otherwise engaged in overkill, possibly revealing their wishes to exact revenge and erase the despised victim from existence.

YPOs, like other murderers, may alter bodies when “staging” a crime scene [11]. Staging behavior is defined as the intentional and purposeful manipulation of the behavioral and/or forensic evidence found at the crime scene [17, 22]. Staging at a crime scene is an effort by the offender to create a new or different scene and a new motive to misdirect the investigation away from himself. The offender attempts to obfuscate what would otherwise be a law enforcement investigator's logical inference regarding what occurred. The offender assumes that without an attempt to redirect the investigation, law enforcement would quickly focus on them as a logical suspect due to a pre‐existing relationship with the victim, the location, or both. The totality of the circumstances of the crime scene must be considered to determine if staging has occurred, and every observable behavior is analyzed and reconciled with the logic of the available forensic evidence. In short, when the crime scene does not make sense, staging should be considered [17, 22].

The aims of this descriptive, retrospective study on YPOs were: (1) to report in‐depth data on their psychopathology, level of psychopathy, and criminal characteristics and (2) to explore the interaction between these characteristics and their crime scene behaviors, with a particular focus on body management. An underlying premise was that biopsychosocial factors like intelligence, personality, presence of mental illness, offender‐victim relationship, family dynamics, and environmental circumstances would influence their crime scene decision‐making after the murders. Three main offender categories, consistent with the YPO literature, were verified for the sample based on interview and case file data: those who had been victims of chronic child abuse, those with a significant history of antisocial behavior, and those who had active psychotic disorders at the time of the murders. Several additional empirically based questions for the offenders were generated based on these typological findings and author experience in evaluating and treating this population. First, we expected that the child abuse victims would be more likely to engage in undoing behaviors given their theoretically insecure‐ambivalent attachment style due to parental maltreatment [23, 24]. This ambivalence presumably would be based on their having experienced both positive and negative parenting (as is often the case in abused children), and it would then be expressed by their prominent feelings of abuse‐related fear and anger leading to murder followed by undoing behaviors stemming from guilt and sadness. Second, we anticipated that youth with elevated levels of psychopathy would be less likely to engage in undoing behaviors due to egocentric, callous, unemotional traits [25, 26], and more likely to transport their victims' bodies away from the crime scene to hide this evidence for the purpose of evading detection by law enforcement. For the youth with psychotic disorders, we thought it likely that their body management behaviors would be disorganized and less predictable due to their delusional, confused mental state [11].

## 2 MATERIALS AND METHODS

### 2.1 The sample and inclusion criteria

The sample was composed of 21 YPOs compiled from the authors' forensic consultation practices (18 cases: reasons for consultation were to assess competency to stand trial, to assess mental state at the time of the offense, and/or for mitigation purposes at the sentencing phase of trial) or research files with extensive data compiled from public information sources (3 cases). All were ≤ 21 at the time of the murders. Six of the 21 youth were ages 18–21. These older youth were living in a parental home and dependent on their parent(s), and thus they were in the late stage of adolescent development (e.g., still residing with one's family of origin, not having achieved financial independence). The age range for defining adolescence has ascended over time, in part due to advances in brain development research, with more recent definitions including children ages 10–24 [27]. In order to assemble a more representative sample of YPOs, inclusion criteria were limited to having committed parricide, being age 21 or less, and not having advanced beyond the adolescent stage of development. There were otherwise no exclusion criteria. The cases were from six states within the United States (20 cases) and one case was from Europe.

### 2.2 Offender assessment

All but three of the 21 youth were clinically interviewed one or more times by one of the authors, and detailed, reliable data were available for all cases (e.g., law enforcement investigation documents, witness statements, crime scene photos, autopsy reports, jail records, school records, pediatric records, mental health records). Psychometric testing performed by the interviewing authors or contained in the records was also relied upon. Coding of the case variables was performed by the authors who consulted on the cases, and then the data was verified by an author independent of the initial coding process. These cases were adjudicated through the adult court system and their case data was thus in the public domain (the analysis of deidentified, publicly available data does not require IRB review).

### 2.3 Psychiatric diagnosis and measurement of psychopathy

Psychiatric diagnoses were derived from interview and case file data using DSM‐5‐TR criteria to achieve best estimate diagnoses [28]. The degree of psychopathy was assessed using the Hare Psychopathy Checklist‐Revised (PCL‐R) [29] for those 18 or older and the Hare Psychopathy Checklist: Youth Version [30] for those under 18. The PCL:YV is a slightly modified version of the 20‐item PCL‐R for use in youth ages 12–18. Both versions are clinician rated scales designed to assess the traditional construct of psychopathy. PCL scoring for the sample was initially done by the author who conducted the clinical interview, and then the PCL scoring was jointly reviewed with at least one and sometimes two other authors during research meetings. When uncertainty or disagreement arose for PCL criteria, which was infrequent, then the case information was discussed amongst the authors to reach 100% rater consensus on scoring for all PCL items. For the purposes of this study and based on previous research methodology [11], PCL scores of <10 were considered in the normal range, scores of 10–19 indicative of a mild degree of psychopathic traits, scores of 20–29 a moderate degree of psychopathic traits, and ≥ 30 a severe degree of psychopathic traits.

### 2.4 Data analysis

Cross‐tabular analyses and Fisher's exact tests (i.e., on cells with less than five expected values) were performed to compare the offender and offending characteristics of YPOs who were victims of chronic child abuse versus nonvictims of child abuse, with elevated PCL‐R scores (≥10) versus those with low PCL scores (<10), and with psychotic symptoms versus those without psychotic symptoms. To evaluate the strength of the relationships, phi (between two variables on two levels on each variable) and Cramer's *V* (between two variables with at least three levels on one variable) coefficients were used. A relationship of 1.00 specifies a perfect relationship. The Cohen's standard for the interpretation of cross‐tabular effect sizes indicates that phi values of 0.10–0.29 were regarded as small effects, 0.30–0.49 were considered as moderate effects, and values of 0.50 and above as strong effects. The effect size for Cramer's V coefficients of 0.16 and below was regarded as weak, values between 0.17 and 0.28 as moderate, and 0.29 and above as strong (see Ref. [31]). When the degrees of freedom exceeded 1 (e.g., a 2 × 3 matrix), the Bonferroni correction method was computed to compare the column proportions. The significance level was set at 0.05.

## 3 RESULTS

### 3.1 Offender demographics, family background, and child abuse history

The mean age of the 21 youthful parricide offenders was 16.7 years (*SD* = 2.7, range = 11–21). Eighteen were male (86%) and most (86%) were White. In terms of the offender's education and intelligence, the majority were in senior high school (62%) at the time of the murders, and the others were in junior high school (29%) or college (9%). All of them fell within the normal IQ range (average, high average, or low average IQ), with most in the high average range, that is, standard score 110–119 (*n* = 13; 62%). None of them were intellectually disabled (formerly known as mental retardation). Slightly over a quarter (*n* = 6; 29%) of the offenders had a history of criminal activity or arrests (see Table 1).

**TABLE 1:** Frequencies of youthful parricide offenders as victims of child abuse (*N* = 21).

	Victim of child abuse					
Variables	All sample	No (*n* = 8)	Yes (*n* = 13)	*χ* ^2^/Fisher's exact test	Phi/Cramer‘s *V*	*p*
	*N* (%)	*N* (%)	*N* (%)			
**Offender demographics**						
Offender age (mean)	16.67	16.38	16.85		*t* = −0.35	0.707
	(*SD* = 2.69)	(*SD* = 3.42)	(*SD* = 2.27)			
Offender sex						
Male	18 (85.7%)	7 (87.5%)	11 (84.6%)	0.03	0.04	1.000
Female	3 (14.3%)	1 (12.5%)	2 (15.4%)			
Offender race						
White	18 (85.7%)	8 (100.0%)	10 (76.9%)	2.15	0.32	0.257
Black	3 (14.3%)	0 (0.0%)	3 (23.1%)			
Victim‐offender relationship						
Father	6 (28.6%)	1 (12.5%)	5 (38.5%)	3.46	0.41	0.177
Mother	7 (33.3%)	2 (25.0%)	5 (38.5%)			
Both parents	8 (38.1%)	5 (62.5%)	3 (23.1%)			
Living condition						
Single‐parent home	8 (33.3%)	1 (12.5%)	6 (46.2%)	2.52	0.35	0.174
Two‐parent home	13 (66.7%)	7 (87.5)	7 (53.8%)			
**Offender intelligence, lifestyle, and mental conditions**						
Highest education level						
Junior high school (G6‐9)	6 (28.6)	4 (50.0%)	2 (15.4%)	3.44	0.40	0.179
Senior high school (G10‐12)	13 (61.9%)	3 (37.5%)	10 (76.9%)			
College/university	2 (9.5%)	1 (12.5%)	1 (7.7%)			
IQ Classification						
High average (110–119)	13 (61.9%)	5 (62.5%)	8 (61.5%)	0.68	0.18	0.711
Average (90–109)	7 (33.3%)	3 (37.5%)	4 (30.8%)			
Low average (80–89)	1 (4.8%)	0 (0.0%)	1 (7.7%)			
Criminal History						
No	15 (71.4%)	4 (50.0%)	11 (84.6%)	2.91	0.37	0.146
Yes	6 (28.6%)	4 (50.0%)	2 (15.4%)			
Parental substance abuse disorder						
No	15 (76.2%)	8 (100.0%)	8 (61.5%)	4.04	0.44	0.111
Yes	6 (23.8%)	0 (0.0%)	5 (38.5%)			
Marijuana use						
No	19 (90.5%)	8 (100.0%)	11 (84.6%)	1.36	0.25	0.505
Yes	2 (9.5%)	0 (0.0%)	2 (15.4%)			
Polysubstance use disorder						
No	18 (85.7%)	5 (62.5%)	13 (100.0%)	5.69	0.52*	0.042
Yes	3 (14.3%)	3 (37.5%)	0 (0.0%)			
ADHD						
No	20 (95.2%)	7 (87.5%)	13 (100.0%)	1.71	0.29	0.381
Yes	1 (4.8%)	1 (12.5%)	0 (0.0%)			
Autism						
No	20 (95.2%)	8 (100.0%)	12 (92.3%)	0.65	0.18	1.000
Yes	1 (4.8%)	0 (0.0%)	1 (7.7%)			
PTSD						
No	15 (71.4%)	8 (100.0%)	7 (53.8%)	5.17	0.50*	0.046
Yes	6 (28.6%)	0 (0.0%)	6 (46.2%)			
Major depression disorder						
No	15 (71.4%)	6 (75.0%)	9 (69.2%)	0.08	0.06	1.000
Yes	6 (28.6%)	2 (25.0%)	4 (30.8%)			
Dysthymia						
No	20 (95.2%)	7 (87.5%)	13 (100.0%)	1.71	0.29	0.381
Yes	1 (4.8%)	1 (12.5%)	0 (0.0%)			
Conduct disorder						
No	17 (81.0%)	6 (75.0%)	11 (84.6%)	0.30	0.12	0.618
Yes	4 (19.0%)	2 (25.0%)	2 (15.4%)			
Antisocial personality disorder						
No	19 (90.5%)	6 (75.0%)	13 (100.0%)	3.59	0.41	0.133
Yes	2 (9.5%)	2 (25.0%)	0 (0.0%)			
Borderline personality disorder						
No	20 (95.2%)	7 (87.5%)	13 (100.0%)	1.71	0.29	0.381
Yes	1 (4.8%)	1 (12.5%)	0 (0.0%)			
Schizotypal personality disorder						
No	18 (81.0%)	6 (75.0%)	11 (84.6%)	0.30	0.12	0.618
Yes	3 (19.0%)	2 (25.0%)	2 (15.4%)			
Schizoid personality disorder						
No	20 (95.2%)	8 (100.0%)	12 (92.3%)	0.65	0.18	1.000
Yes	1 (4.8%)	0 (0.0%)	1 (7.7%)			
**Modus operandi**						
Offender motivation						
Abuse	9 (42.9%)	0 (0.0%)^a^	9 (69.2%)^a^	9.85	0.69**	0.007
Anger and resentment	7 (33.3%)	5 (62.5%)	2 (15.4%)			
Psychotic symptoms	5 (23.8%)	3 (37.5%)	2 (15.4%)			
Murder weapon group						
Strangulation	1 (4.8%)	0 (0.0%)	1 (7.7%)	3.33	0.40	0.343
Edged weapon	5 (19.0%)	1 (12.5%)	3 (23.1%)			
Bludgeon	6 (19.0%)	3 (37.5%)	1 (7.7%)			
Firearm	12 (57.1%)	4 (50.0%)	8 (61.5%)			
Plan to avoid arrest						
No	11 (52.4%)	4 (50.0%)	7 (53.8%)	0.03	0.04	1.000
Yes	10 (47.6%)	4 (50.0%)	6 (46.2%)			
Note left at crime scene						
No	17 (81.0%)	6 (75.0%)	11 (84.6%)	0.30	0.12	0.618
Yes	4 (19.0%)	2 (25.0%)	2 (15.4%)			
Victim moved after murder						
No	12 (61.9%)	5 (62.5%)	8 (61.5%)	0.01	0.01	1.000
Yes	9 (38.1%)	3 (37.5%)	5 (38.5%)			
Victim altered after murder						
No	9 (47.6%)	4 (50.0%)	6 (46.2%)	0.03	0.04	1.000
Yes	12 (52.4%)	4 (50.0%)	7 (53.8%)			
Victim transported away from home after murder						
No	18 (57.1%)	4 (50.0%)	8 (61.5%)	0.27	0.11	0.673
Yes	3 (42.9%)	4 (50.0%)	5 (38.5%)			

^a^ Indicated significant differences.

**p < 0.01; *p < 0.05.

Thirteen of the youth (62%) lived in a two‐parent home at the time of the murders; the biological parents were in eight (38%) of these homes, a biological and stepparent were in four homes (19%), and adoptive parents were in one home (5%). Eight of the youth (38%) lived in a single parent home.

About two‐thirds of the offenders (*n* = 13; 62%) experienced one or more forms of chronic child abuse: emotional abuse (62%) was the most frequent, followed by physical abuse (57%) and sexual abuse (19%). These figures exceed 100% as there was overlap in abuse type for most youth, with emotional and physical abuse co‐occurring in 12 of the 13 child abuse cases (92%). Parental substance abuse was present in six of the homes (29%) where the parricides occurred. There was a nonsignificant trend for offenders who lived in two‐parent homes to be less likely to have experienced child abuse (*n* = 6; 46%) than those in one‐parent homes (*n* = 7; 88%).

### 3.2 DSM‐5 psychiatric diagnoses

Nearly the entire sample (*n* = 19; 95%) met criteria for at least one DSM diagnosis. The most prevalent psychiatric diagnoses in the offenders were PTSD (*n* = 6; 29); major depressive disorder (*n* = 5; 24%); any psychotic disorder (*n* = 5; 24%—two had major depressive disorder with psychotic features, and one each had schizophrenia, schizoaffective disorder, and brief psychotic disorder); conduct disorder (*n* = 4; 19%); and any substance use disorder (*n* = 4; 19%—polysubstance use disorder was present in three and marijuana use disorder in one). Personality disorders were present in six (30%) of the sample: three had schizotypal personality disorder (14%), one had antisocial personality disorder (5%), one had combined antisocial and borderline personality disorder (5%), and one had schizoid personality disorder (5%). Those who were suffered chronic child abuse were significantly more likely to have a PTSD Diagnosis (Fisher's exact test = 5.17, *p* = 0.046, phi = 0.50).

### 3.3 Crime characteristics

All the parricidal acts occurred in the family home. In three cases (14%), the offenders left the family home after murdering their parent(s) and killed additional victims: two youth went to their school and killed students, and the third youth went into the community and killed victims. These three parricides, during the second phase of their homicidal behavior, evolved into spree murders, defined as someone who commits murders in two or more locations within a short period of time [32].

The most oft‐used murder methods, in descending order, were firearms (*n* = 12; 57%), bludgeons (*n* = 6; 29%), bladed weapons (*n* = 5; 24%), and asphyxiation (*n* = 1; 5%). These figures exceed 100% because in three cases two types of weapons were used. Offenders with psychosis were significantly more likely to use bladed weapons (60%) to kill their victim, while those without psychotic symptoms tended to use firearms (62.5%) (Fisher's exact test = 7.68, *p* = 0.049). The strength of this relationship was strong (Cramer's *V* = 0.60) (see Table 2).

**TABLE 2:** Frequencies of youthful parricide offenders with high PCL‐R score and psychotic symptoms (*N* = 21).

	High PCL‐R score					Psychotic symptoms				
Variables	No (*n* = 12)	Yes (*n* = 9)	*χ* ^2^/Fisher's	Phi/*p* exact test	Cramer's *V*	No (*n* = 12)	Yes (*n* = 9)	*χ* ^2^/Fisher's exact test	Phi/Cramer‘s *V*	*p*
	*N* (%)	*N* (%)				*N* (%)	*N* (%)			
**Offender demographics**										
Offender age (mean)	16.75	16.56		*t* = 0.16	0.875	16.94	15.80		*t* = 0.56	0.605
	(*SD* = 3.08)	(*SD* = 2.24)				(*SD* = 2.02)	(*SD* = 4.44)			
Offender sex										
Male	10 (83.3%)	8 (88.9%)	0.13	0.08	1.000	14 (87.5%)	4 (80.0%)	0.18	0.09	1.000
Female	2 (16.7%)	1 (11.1%)				2 (12.5%)	1 (20.0%)			
Offender race										
White	9 (75.0%)	9 (100.0%)	2.63	0.35	0.229	14 (87.5%)	4 (80.0%)	0.18	0.09	1.000
Black	3 (25.0%)	0 (0.0%)				2 (12.5%)	1 (20.0%)			
Victim‐offender relationship										
Father	4 (33.3%)	2 (22.2%)	2.07	0.31	0.356	4 (25.0%)	2 (40.0%)	0.95	0.21	0.621
Mother	5 (41.7%)	2 (22.2%)				5 (31.3%)	2 (40.0%)			
Both parents	3 (25.0%)	5 (55.6%)				7 (43.8%)	1 (20.0%)			
Living condition										
Single‐parent home	5 (41.7%)	2 (22.2%)	0.87	0.20	0.642	5 (31.3%)	2 (40.0%)	0.13	0.08	1.000
Two‐parent home	7 (58.3%)	7 (77.8%)				11 (68.7%)	3 (60.0%)			
**Offender intelligence, lifestyle, and mental conditions**										
Highest education level										
Junior high school (G6‐9)	3 (25.0%)	3 (33.3%)	1.68	0.28	0.431	3 (18.8%)	3 (60.0%)	4.89	0.48^+^	0.087
Senior high school (G10‐12)	7 (58.3%)	6 (66.7%)				12 (75.0%)	1 (20.0%)			
College/university	2 (16.7%)	0 (0.0%)				1 (6.3%)	1 (20.0%)			
IQ Classification.										
High average (110–119)	8 (66.7%)	5 (55.6%)	1.44	0.26	0.488	8 (50.0%)	5 (100.0%)	4.04	0.44	0.133
Average (90–109)	3 (25.0%)	4 (44.4%)				7 (43.8%)	0 (0.0%)			
Low average (80–89)	1 (8.3%)	0 (0.0%)				1 (6.3%)	0 (0.0%)			
Criminal History										
No	12 (100.0%)	3 (33.3%)	11.20	0.73**	0.002	10 (62.5%)	5 (100.0%)	2.63	0.35	0.262
Yes	0 (0.0%)	6 (66.7%)				6 (37.5%)	0 (0.0%)			
Parental substance abuse disorder										
No	9 (75.0%)	7 (77.8%)	0.02	0.03	1.000	13 (81.3%)	3 (60.0%)	0.95	0.21	0.634
Yes	3 (25.0%)	2 (22.2%)				3 (18.8%)	2 (40.0%)			
Marijuana use										
No	12 (100.0%)	7 (77.8%)	2.95	0.37	0.171	14 (87.5%)	5 (100.0%)	0.69	0.18	1.000
Yes	0 (0.0%)	2 (22.2%)				2 (12.5%)	0 (0.0%)			
Polysubstance use disorder										
No	12 (100.0%)	6 (66.7%)	4.67	0.47^+^	0.063	13 (81.3%)	5 (100.0%)	1.09	0.23	0.549
Yes	0 (0.0%)	3 (33.3%)				3 (18.8%)	0 (0.0%)			
ADHD										
No	12 (100.0%)	8 (88.9%)	1.40	0.26	0.429	15 (93.8%)	5 (100.0%)	0.33	0.13	1.000
Yes	0 (0.0%)	1 (11.1%)				1 (6.3%)	0 (0.0%)			
Autism										
No	11 (91.7%)	9 (100.0%)	0.79	0.19	1.000	15 (93.8%)	5 (100.0%)	0.33	0.13	1.000
Yes	1 (8.3%)	0 (0.0%)				1 (6.3%)	0 (0.0%)			
PTSD										
No	8 (66.7%)	7 (77.8%)	0.31	0.12	0.659	10 (62.5%)	5 (100.0%)	2.63	0.35	0.262
Yes	4 (33.3%)	2 (22.2%)				6 (37.5%)	0 (0.0%)			
Major depression disorder										
No	8 (66.7%)	7 (77.8%)	0.31	0.12	0.659	12 (75.0%)	3 (60.0%)	0.42	0.14	0.639
Yes	4 (33.3%)	2 (22.2%)				4 (25.0%)	2 (40.0%)			
Dysthymia										
No	11 (91.7%)	9 (100.0%)	0.79	0.19	1.000	16 (100.0%)	4 (80.0%)	3.36	0.40	0.238
Yes	1 (8.3%)	0 (0.0%)				0 (0.0%)	1 (20.0%)			
Conduct disorder										
No	12 (100.0%)	5 (55.6%)	6.59	0.56*	0.021	12 (75.0%)	5 (100.0%)	1.54	0.27	0.532
Yes	0 (0.0%)	4 (44.4%)				4 (25.0%)	0 (0.0%)			
Antisocial personality disorder										
No	12 (100.0%)	7 (77.8%)	2.95	0.37	0.171	14 (87.5%)	5 (100.0%)	0.69	0.18	1.000
Yes	0 (0.0%)	2 (22.2%)				2 (12.5%)	0 (0.0%)			
Borderline personality disorder										
No	12 (100.0%)	8 (88.9%)	1.40	0.26	0.429	15 (93.8%)	5 (100.0%)	0.33	0.13	1.000
Yes	0 (0.0%)	1 (11.1%)				1 (6.3%)	0 (0.0%)			
Schizotypal personality disorder										
No	11 (91.7%)	6 (66.7%)	2.08	0.32	0.272	13 (81.3%)	4 (80.0%)	0.01	0.01	1.000
Yes	1 (8.3%)	3 (33.3%)				3 (18.7%)	1 (20.0%)			
Schizoid personality disorder										
No	11 (91.7%)	9 (100.0%)	0.79	0.19	1.000	15 (93.8%)	5 (100.0%)	0.33	0.13	1.000
Yes	1 (8.3%)	0 (0.0%)				1 (6.2%)	0 (0.0%)			
**Modus operandi**										
Offender motivation										
Abuse	7 (58.3%)	2 (22.2%)	14.65	0.84**	0.001	9 (56.3%) ^a^	0 (0.0%) ^a^	21.00	1.00***	<0.001
Anger and resentment	0 (0.0%) ^a^	7 (77.8%) ^b^				7 (43.8%) ^b^	0 (0.0%) ^b^			
Psychotic symptoms	5 (41.7%) ^c^	0 (0.0%) ^d^				0 (0.0%) ^c^	5 (100.0%) ^c^			
Murder weapon group										
Strangulation	0 (0.0%)	1 (11.1%)	3.99	0.44	0.263	1 (6.3%)	0 (0.0%)	7.68	0.60*	0.053
Edged object	3 (25.0%)	1 (11.1%)				1 (6.3%)	3 (60.0%)			
Blunt object	1 (8.3%)	3 (33.3%)				4 (25.0%)	0 (0.0%)			
Firearm	8 (66.7%)	4 (44.4%)				10 (62.5%)	2 (40.0%)			
Plan to avoid arrest										
No	8 (66.7%)	3 (33.3%)	2.29	0.33	0.198	7 (43.8%)	4 (80.0%)	2.01	0.31	0.312
Yes	4 (33.3%)	6 (66.7%)				9 (56.3%)	1 (20.0%)			
Note left at crime scene										
No	12 (100.0%)	5 (55.6%)	6.59	0.56*	0.021	12 (75.0%)	5 (100.0%)	1.54	0.27	0.532
Yes	0 (0.0%)	4 (44.4%)				4 (25.0%)	0 (0.0%)			
Victim moved after murder (undoing)										
No	9 (75.0%)	4 (44.4%)	2.04	0.31	0.203	8 (50.0%)	5 (100.0%)	4.04	0.44	0.111
Yes	3 (25.0%)	5 (55.6%)				8 (50.0%)	0 (0.0%)			
Victim being altered after murder										
No	8 (66.7%)	2 (22.2%)	4.07	0.44^+^	0.080	5 (31.3%)	5 (100.0%)	7.22	0.59*	0.015
Yes	4 (33.3%)	7 (77.8%)				11 (68.8%)	0 (0.0%)			
Victim moved out of home after murder										
No	10 (83.3%)	2 (22.2%)	7.84	0.61**	0.009	7 (43.8%)	5 (100.0%)	4.92	0.48*	0.045
Yes	2 (16.7%)	7 (77.8%)				9 (56.3%)	0 (0.0%)			

*Note*: a, b, and c indicated significant differences.

**p < 0.01; *p < 0.05; ^+^ *p* < 0.10.

### 3.4 Victims

The victims were both parents in nine cases (43%), and they included six sets of biological parents, two stepparents, and one set of adoptive parents. In the other 12 cases (57%), a single parent was killed: the father in six cases (33%) and the mother in six cases (33%) (one was a grandmother but a de facto mother figure). In five of the cases (24%), victims other than parents were also killed. Other victims killed included three siblings (sister, brother, half‐brother), one grandmother (in addition to the aforementioned one), friends, classmates, a former partner, and strangers.

### 3.5 Plans to avoid detection and crime scene staging

Ten of the youth (48%) had a plan to avoid being caught for their murderous acts. In three cases (14%) the offenders staged the crime scene. In two cases, breaking and entering by an intruder was staged and in both of these cases, the offenders fabricated false evidence of an intruder having broken in through a window. The home was set on fire in one case to destroy evidence, and one youth informed police he saw a masked intruder leaving the home. In the third case, a youth staged hearing the parent being killed while pretending in front of a witness that they were talking on the phone at the time of the deadly attack.

There were a variety of plans to escape blame in the other seven cases. One youth successfully fled to another country but was arrested there and extradited back to the United States for prosecution. Five other youth described plans to flee from their cities and towns and start new lives elsewhere, and their plans were in various stages of development when they were arrested. One youth told police the gun they were holding accidently went off and killed the father who was in the shower; however, this story did not hold water as the totality of the circumstances indicated a purposeful attack rather than an accident.

### 3.6 Crime scene notes

Four youth (19%) left a note at the scene. In one case the note was from an imaginary intruder, and it stated the youth was forced to participate in the crime. In a second case the youth left a note saying they had to kill people, did not know why, and had no other choice. Another offender left a note saying they did not murder the family out of selfishness but because they did not want to die alone when ending their life by suicide (which never actually occurred). In the fourth case, the youth left a manifesto of sorts explaining they were not insane but angry, had never been loved by anyone, and were tired of being mistreated by the world. Offenders with elevated PCL scores (44.4% vs. 0%) were significantly more likely to leave a note at the crime scene (Fisher's exact test = 6.59, *p* = 0.021), and the difference was strong in its effect (phi = 0.56).

### 3.7 Psychopathology and homicidal motivation

Three main offender psychopathology categories were apparent in the sample: those who were victims of chronic emotional, physical, and/or sexual child abuse (*n* = 13; 62%); those with an elevated degree of psychopathic traits (PCL score ≥ 10) (*n* = 9; 43%), and those who had active psychotic disorders at the time of the murders (*n* = 5; 24%). There was overlap between these categories in only six cases (29%): In four of these six cases youths had elevated psychopathic traits and a history of child abuse, and in two cases youths with a psychotic disorder had a history of child abuse. None of the youth with psychotic disorders had elevated psychopathy scores.

Regarding motivation, nine of the offenders (43%) murdered their victims in the setting of chronic child abuse, seven (33%) were primarily motivated by anger and/or a sense of resentment, and five (24%) committed their acts due to psychosis. Common reasons for the youth to kill while angry/resentful were that they perceived parental rules or restrictions to be untenable, frustration over parents not giving them money or possessions they felt entitled to, in reaction to intense arguments, or a combination of the above.

Not unexpectedly, youth with a history of child abuse were significantly more likely to be motivated by their victimization experience than those who were not victims of child abuse. Conversely, offenders who were not child abuse victims were more likely to be motivated by their sense of anger/resentment or psychotic symptoms (Fisher's exact test = 9.85, *p* = 0.007, Cramer's *V* = 0.69). This relationship was strong in effect.

When comparing youthful parricide offenders with elevated (≥ 10) and normal (< 10) PCL scores, five significant differences were observed (see Table 2). Offenders with a high PCL score were significantly more likely than those with a low PCL score to have a criminal history (66.7% vs. 0%; Fisher's exact test = 11.20, *p* = 0.002) and to be diagnosed with conduct disorder (44.4% vs. 0%; Fisher's exact test = 6.59, *p* = 0.021). The strength of both relationships was strong (phi = 0.73 and 0.56, respectively). Offenders with a low PCL score were significantly more likely than their counterparts with a high PCL score to be motivated by being victims of child abuse (58.3% vs. 22.2%) and psychotic symptoms (41.7% vs. 0.0%), whereas those offenders with a high PCL score were more likely to be motivated by their sense of anger/resentment than their counterparts (77.8% vs. 14.3%; Fisher's exact test = 14.65, *p* = 0.001). The effect of this relationship was strong (Cramer's *V* = 0.84).

### 3.8 Psychopathology and body management (body movement and body alteration)

#### 3.8.1 Body movement

Offenders moved bodies to any extent in nine cases (43%). Six of them moved bodies within the family home (29%): five moved bodies to other rooms or home areas and one moved his mother's body to her bedroom floor. Three youth transported bodies away from the family home (14%). In these transportation cases, one youth superficially buried family members on a distant part of the family property, one youth left the father's body inside his car trunk at a school parking lot, and one youth put the father's body in an outdoor shed in a large plastic bin.

Significantly more offenders with a high PCL score than their counterparts with a low PCL score (77.8% vs. 16.7%) moved victim bodies following the murders (Fisher's exact test = 7.84, *p* = 0.009). The effect size of this difference was strong (phi = 0.61). None of the offenders with psychosis altered their victims' bodies (0% vs. 68.8%; Fisher's exact test = 7.22, *p* = 0.015) or moved them out of the house (0% vs. 56.3%; Fisher's exact test = 4.92, *p* = 0.045). A moderate to strong association was found in these relationships (phi = 0.59 and 0.48, respectively).

#### 3.8.2 Body alteration

There was evidence of body alteration in 12 of the cases (57%). The most observed type of body alteration was what would be considered undoing, present in seven cases (33%). In five of these cases, the parents' bodies were covered in sheets or bedding, and in the other two cases their faces were covered with a pillow. The motivation of these seven youths was confirmed through interviews and case data as classic undoing (e.g., due to feelings of sorrow or guilt, to comfort the victim, to help deny the reality of the murder). The magical thinking aspect of undoing behavior is illustrated by a youth who put the father's body in a bin and covered him with a blanket for warmth before moving his body outside to a shed during the winter. In the other five cases, the body alterations were for practical purposes. In three of these cases, the bodies were covered for concealment purposes, in one case the body was wrapped in bedding to make transportation easier, and in one case the body was set on fire as part of staging a breaking and entering crime by a concocted homicidal intruder.

## 4 DISCUSSION

This descriptive, retrospective study of 21 youthful parricide offenders has reported in‐depth data on their biopsychosocial and criminal characteristics, which included psychopathology, level of psychopathic traits, and their relationship to crime scene actions. Studies examining the influence of mental illness and psychopathic tendencies on parricidal behaviors by youth are scarce, and to our knowledge this is the largest study to date examining this topic. While this sample was not randomly selected, these youth shared common characteristics with juvenile parricide offenders in that most of them killed one parent, were victims of chronic child abuse, met diagnostic criteria for one or more psychiatric diagnoses, used a firearm, and committed the killings at the family home [11, 33]. Also consistent with the literature on typology was that these 21 youths fell fairly cleanly into the three established categories of parricidal motivation, that is, being chronic child abuse victims, having marked conduct disorder/antisocial traits, or having serious mental illness. Limited overlap between these categories existed, and this phenomenon would generally be expected with any classification system given the complexities of psychopathology and human behavior. And, as might be expected, the most common DSM diagnosis in the sample was PTSD, and those who suffered chronic child abuse were significantly more likely to have a PTSD diagnosis as a result.

Only one of our three questions concerning the interaction between psychopathology and body management was supported, and not entirely. First, the child abuse victims were not more likely to engage in undoing behaviors than the non‐abused youth. There was only a nonsignificant trend for the abused youth to perform undoing behaviors (71%) compared to those who were not abused (29%). Second, we expected youth with elevated PCL scores would be less likely to show undoing behaviors and more likely to transport their victims' bodies away from the crime scene to try to avoid culpability. However, no difference was found in the seven youth who engaged in undoing regarding their PCL scores. In fact, contrary to our expectations, four of the seven youth (57%) with undoing behavior had elevated PCL scores in the moderate to high zone, ranging from 21 to 30 with a mean of 25.3. A nearly significant trend (*p* < 0.06) for the second supposition was seen as all three of the offenders who transported bodies from the crime scene had notably elevated psychopathic traits, ranging from 19 to 27 with a mean of 23.3, whereas none of the 13 youth with normal PCL scores transported bodes from the crime scene. And significantly more offenders with an elevated PCL score compared with their counterparts with a low PCL score (77.8% vs. 16.7%) moved victim bodies to any extent following the murders. For the youth with psychosis, we anticipated that their body management behaviors would be disorganized and less predictable due to their delusional, confused mental state. In fact, their post‐crime behaviors were quite consistent in that none of the offenders with psychosis engaged in undoing behaviors, altered victim bodies, or moved them either within or away from the home. Furthermore, none of them youth had elevated PCL scores. They also most often killed using bladed weapons, whereas the nonpsychotic youth usually used firearms.

A particular strength of this study was that detailed body management information was available for this sample, an area that has rarely been considered in previous YPO research. We found that body alteration occurred in over half the cases and undoing was the most common form of body alteration, present in one‐third of the sample. The limited prior research on undoing has tended to show that it is rarely present at homicide crime scenes. It being so prevalent in this study is most likely because these cases involved children killing parents. Thus, in every case there was the influence of parent–child attachments and these perpetrators having been raised by the victim(s). Through post‐crime clinical interviews, the youth confirmed their motivations for undoing, which was typically a combination of guilt, sadness, wanting to symbolically comfort their parent, and/or trying to deny the reality that they had committed murder.

As can be seen by the present findings and previous literature, the motivation for body alteration has numerous other explanations besides undoing [18, 34, 35]. For example, body alteration may also be driven by efforts to conceal victims from witnesses; the need to wrap the body in order to facilitate handling and transportation of it; the desire for revenge or the catharsis of rage which may culminate in excessive violence or overkill; delusional thinking related to psychotic disorders with potentially unusual or bizarre bodily findings; staging of the crime scene; and, while extremely rare, paraphilic gratification. Importantly, offender motivation for engaging in body alteration may not be understood through crime scene findings alone and will only be understood once post‐arrest interviews are conducted.

Other notable findings were apparent in the sample. Three‐fifths of these youth were in the high average range of intelligence (62%). In general, juvenile murderers have been found to have lower than average IQs [36, 37]. Although the literature on the correlation between IQ and violence is inconsistent, some research suggests higher intelligence moderates the relationship between juvenile aggression and total psychopathy, with the highest offending levels exhibited by youth with relatively greater levels of psychopathy and IQ [38]. Perhaps higher intelligence in the YPOs in this study contributed to their ability to successfully plan and complete the act of killing a parent. While this is an intriguing finding, it is beyond the scope of our study to draw any solid inferences in this regard.

Slightly more than one‐quarter (29%) of the sample had a history of criminal activity or arrests, and they all also had diagnoses of either conduct disorder (four) or antisocial personality disorder (two). At least for this sample, the preexisting history of criminality or prior arrests or one of these two diagnoses would not have been particularly useful as risk factors for impending parricide, as these variables were not present in most of the sample and none of the preexisting antisocial behaviors involved violence toward a parent.

As noted in the results, personality disorder diagnoses were present in almost one‐third of the sample, and four of the 21 (19%) youth had either schizotypal or schizoid personality disorder (from the DSM “Cluster A” grouping of personality disorders, those with “odd, eccentric” features). Moreover, the three youth who committed post‐parricide spree murderers had either schizotypal (two) or schizoid personality disorder (one). These latter two diagnoses have been found to be overrepresented in prior juvenile and adult homicide offender populations [39, 40].

About half of the youth revealed or demonstrated plans to avoid culpability for the homicides, which varied from staging the crime scene to notions to travel far away not yet acted upon to sophisticated and temporarily successful plans, for example, being arrested in another country. There was no relationship found between elevated PCL scores and plans to avoid responsibility, although the trend existed in which the four youth who left notes at the crime scene had elevated PCL scores and each note to some degree contained exculpatory information. Other findings in the YPOs with elevated PCL scores were their being significantly more likely to have a criminal history, to be diagnosed with conduct disorder, and to be motivated to kill by a sense of anger/resentment. In contrast, youth with low PCL scores were most likely to be motivated by being victims of child abuse.

Several main limitations pertain to this research project. First, the sample examined was one of convenience and may not be representative of YPOs. Second, the sample was relatively small, and thus a deeper analysis of possible associations between psychopathology and crime scene behaviors was not possible. However, research on parricide is inherently saddled with methodological challenges, like low base rates and small case cohorts, that make it difficult to obtain generalizable findings [10, 41]. Further, while criminal databases exist with access to larger samples of parricide offenders, such as the FBI Supplementary Homicide Reports (SHR), they suffer from a lack of detail beyond basic information on demographics and crime characteristics.

In summary, research on the relationship between mental disorders, psychopathy level, and crime scene behaviors is nascent and in need of further investigation. This study has offered only a preliminary foray into this area, and in many ways it raises more questions than answers for this population. One goal of future research should be to help clarify which combinations of risk factors signal the greatest risk for impending parricide—for example, type and degree of mental illness, personality disturbances, history of child abuse, disturbed family dynamics—so that proactive interventions can be implemented with at‐risk families before the tragedy of parricide strikes. Additionally, we hope this study and future related research will help law enforcement investigators and forensic mental health professionals better understand the significance of body management behaviors, like undoing and body movement, and what these and other crime scene actions can tell us about these youthful perpetrators.

## CONFLICT OF INTEREST STATEMENT

The authors have no conflicts of interest to report.
